# A TGF‐β signaling‐related lncRNA signature for prediction of glioma prognosis, immune microenvironment, and immunotherapy response

**DOI:** 10.1111/cns.14489

**Published:** 2023-10-18

**Authors:** Wei‐Wei Duan, Li‐Ting Yang, Jian Liu, Zi‐Yu Dai, Ze‐Yu Wang, Hao Zhang, Xun Zhang, Xi‐Song Liang, Peng Luo, Jian Zhang, Zao‐Qu Liu, Nan Zhang, Hao‐Yang Mo, Chun‐Run Qu, Zhi‐Wei Xia, Quan Cheng

**Affiliations:** ^1^ Department of Neurosurgery, Xiangya Hospital Central South University Changsha Hunan China; ^2^ National Clinical Research Center for Geriatric Disorders, Xiangya Hospital Central South University Changsha Hunan China; ^3^ Department of Neurology, Xiangya Hospital Central South University Changsha Hunan China; ^4^ Experiment Center of Medical Innovation The First Hospital of Hunan University of Chinese Medicine Changsha Hunan China; ^5^ MRC Centre for Regenerative Medicine, Institute for Regeneration and Repair University of Edinburgh Edinburgh UK; ^6^ Department of Oncology, Zhujiang Hospital Southern Medical University Guangzhou China; ^7^ Department of Interventional Radiology The First Affiliated Hospital of Zhengzhou University Zhengzhou Henan China; ^8^ One‐third Lab, College of Bioinformatics Science and Technology Harbin Medical University Harbin Hei Longjiang China; ^9^ Department of Neurology Hunan Aerospace Hospital Changsha Hunan China

**Keywords:** glioma, immune infiltration, immunotherapy, lncRNA, prognosis, TGF‐β

## Abstract

**Aims:**

The dysregulation of TGF‐β signaling is a crucial pathophysiological process in tumorigenesis and progression. LncRNAs have diverse biological functions and are significant participants in the regulation of tumor signaling pathways. However, the clinical value of lncRNAs related to TGF‐β signaling in glioma is currently unclear.

**Methods:**

Data on glioma's RNA‐seq transcriptome, somatic mutation, DNA methylation data, and clinicopathological information were derived from the CGGA and TCGA databases. A prognostic lncRNA signature was constructed by Cox and LASSO regression analyses. TIMER2.0 database was utilized to deduce immune infiltration characteristics. “ELMER v.2” was used to reconstruct TF‐methylation‐gene regulatory network. Immunotherapy and chemotherapy response predictions were implemented by the TIDE algorithm and GDSC database, respectively. In vitro and in vivo experiments were conducted to verify the results and clarify the regulatory mechanism of lncRNA.

**Results:**

In glioma, a TGF‐β signaling‐related 15‐lncRNA signature was constructed, including AC010173.1, HOXA‐AS2, AC074286.1, AL592424.1, DRAIC, HOXC13‐AS, AC007938.1, AC010729.1, AC013472.3, AC093895.1, AC131097.4, AL606970.4, HOXC‐AS1, AGAP2‐AS1, and AC002456.1. This signature proved to be a reliable prognostic tool, with high risk indicating an unfavorable prognosis and being linked to malignant clinicopathological and genomic mutation traits. Risk levels were associated with different immune infiltration landscapes, where high risk was indicative of high levels of macrophage infiltration. In addition, high risk also suggested better immunotherapy and chemotherapy response. cg05987823 was an important methylation site in glioma progression, and AP‐1 transcription factor family participated in the regulation of signature lncRNA expression. AGAP2‐AS1 knockdown in in vitro and in vivo experiments inhibited the proliferation, migration, and invasion of glioma cells, as well as the growth of glioma, by downregulating the expression levels of NF‐κB and ERK 1/2 in the TGF‐β signaling pathway.

**Conclusions:**

A prognostic lncRNA signature of TGF‐β signaling was established in glioma, which can be used for prognostic judgment, immune infiltration status inference, and immunotherapy response prediction. AGAP2‐AS1 plays an important role in glioma progression.

## Graphical abstract

A TGF‐β signaling‐related lncRNA signature has been established in glioma, which can be used for prognostic judgment, immune infiltration status speculation, and immunotherapy response prediction. Furthermore, in vitro experiments confirmed that silencing of TGF‐β signaling‐related lncRNA could inhibit the proliferation of glioma cells. This study provides candidate biomarkers with clinical application value for glioma.

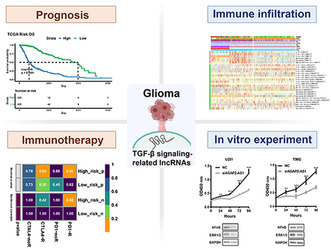

## 1 INTRODUCTION

As the primary brain tumor with the highest incidence,^1^ glioma has a poor prognosis. The average survival time for low‐grade glioma (LGG) is approximately 7 years,^2^ and the median survival time for glioblastoma (GBM) is <2 years.^3^ The WHO classification of glioma based on histological characteristics can reflect the degree of malignancy and prognosis to a certain extent.^4^ However, histopathologic classification suffers from high observer variability, and certain histological entities include a range of tumor groups that differ significantly in biology, therapeutic response, and prognosis.^1^, ^5^, ^6^ Hence, finding new biomarkers and therapeutic targets holds great clinical importance in refining glioma classification and enhancing prognosis.

The transforming growth factor‐β (TGF‐β) signaling pathway regulates a variety of critical events in cell proliferation, differentiation, and the immune system.^7^ Its perturbation is implicated in cancer pathogenesis, which promotes tumorigenesis and metastasis.^8^, ^9^ Active TGF‐β signaling has been identified in high‐grade gliomas and is associated with poor outcomes. Its carcinogenic effect in glioma is pleiotropic, including induction of proliferation, invasion, angiogenesis, immunosuppression, and promotion of glioma‐initiating cells self‐renewal.^10^, ^11^

Long non‐coding RNAs (lncRNAs), which are longer than 200 nucleotides, do not have the ability to code.^12^ Nevertheless, they engage in a diverse array of cell signaling pathways and biological functions. Their disturbances are implicated in disease and tumor pathology.^13^, ^14^ In gliomas, aberrant lncRNA expression is related to the malignant phenotype, histological differentiation, and prognosis.^15^ LncRNA RMRP is highly expressed in glioma, contributing to the progression of glioma through its impact on the Wnt/β‐catenin signaling.^16^ SNHG16 plays a carcinogenic role by activating PI3K/AKT pathway in glioma.^17^

Given the pivotal roles of TGF‐β signaling and lncRNAs in glioma biology, this study utilized the Chinese Glioma Genome Atlas (CGGA)^18^ and The Cancer Genome Atlas (TCGA, http://cancergenome.nih.gov/) databases to comprehensively analyze glioma data, constructing a TGF‐β signaling‐related lncRNA (TSRlncRNA) signature. This signature was an excellent predictive tool for prognosis and therapeutic response and correlated with prominent tumor hallmarks. Additionally, we conducted in vitro and in vivo validation experiments and explored the potential regulatory mechanism of TSRlncRNA. Our research provides candidate biomarkers with clinical application value for glioma.

## 2 MATERIALS AND METHODS

### 2.1 Data sources

Glioma RNA‐seq transcriptome and clinical data were derived from CGGA^18^ and TCGA databases. A total of 645 samples obtained from TCGA database were included in this study and served as the training set. A total of 306 samples obtained from CGGA database were used as the validation set. Pan‐cancer data including 33 cancer types were acquired using TCGA database. Abbreviations for the 33 cancer types are summarized in Table S1.

### 2.2 Screening of prognostic TSRlncRNAs

We first collected a gene set related to the TGF‐β signaling pathway, encompassing 43 core genes that regulate or mediate TGF‐β signaling.^19^ TSRlncRNAs were obtained through correlation analysis with a threshold of |correlation coefficient| > 0.5 and *p* < 0.05. Subsequently, Cox regression analyses were performed to screen out survival‐related lncRNAs preliminarily. Finally, TSRlncRNAs with the most prognostic value were acquired by the least absolute shrinkage and selection operator (LASSO) regression. The miRTarBase^20^ and starBase^21^ databases were used for the prediction of miRNA‐mRNA and lncRNA‐miRNA interactions to construct competing endogenous RNAs (ceRNA) network, which was visualized by Cytoscape software. The Maximal Clique Centrality algorithm was applied for the determination of hub genes. In addition, the Gene Ontology (GO) analysis^22^ for functional annotation was conducted using the “clusterProfiler” R package.^23^

### 2.3 Consensus clustering analysis

To identify glioma TSRlncRNA subtypes, we utilized the “ConsensusClusterPlus” R package for unsupervised consensus clustering (resample rate of 80% and 1000 iterations). The maximum evaluated k was ten, and the optimal k was determined by inspecting the ConsensusClusterPlus output.^24^ Through principal component analysis (PCA), the mRNA expression data underwent dimensionality reduction to validate distinctions between subtypes.

### 2.4 Establishment of risk scoring model

The risk model based on the TSRlncRNA signature was established as follows:

$$\text{Risk score}={\text{expr}}_{1}\times \beta_{1}+{\text{expr}}_{2}\times \beta_{2}+\cdots +{\text{expr}}_{n}\times \beta_{n}$$

expr is the lncRNA expression value, *β* represents the LASSO regression coefficient, and *n* is the number of the signature lncRNAs. To assess the model's ability to discriminate, the analysis of the receiver operating characteristic (ROC) curve was employed. The construction of the nomogram was done with the “rms” R package. The calibration curves were generated to evaluate the performance of the nomogram.

### 2.5 Genome mutation and immune infiltration analysis

Using the “maftools” R package,^25^ we analyzed and visualized TCGA glioma somatic mutation data. TIMER2.0 database^26^ integrating multiple immune deconvolution algorithms was utilized to estimate immune infiltration features. Gene set variation analysis (GSVA)^27^ based on 24 immune cell marker genes^28^ was performed to quantify immune cells in the samples.

### 2.6 Immunotherapy and chemotherapy response prediction

The prediction of response to immune checkpoint blockade therapy utilized the Tumor Immune Dysfunction and Exclusion (TIDE),^29^ an algorithm that simulates tumor immune evasion mechanisms. The glioma cell line data from the Genomics of Drug Sensitivity in Cancer (GDSC) database^30^ were utilized for the prediction of chemosensitivity. The half‐maximal inhibitory concentration (IC_50_) was calculated utilizing the “pRRophetic” R package.

### 2.7 Construction of TF‐methylation‐gene regulatory network

The TCGA database provided glioma DNA methylation data, which were analyzed using “ELMER v.2”,^31^ a tool inferring transcription factor (TF) networks and regulatory element landscapes from cancer methylomes. Thresholds of *p* < 0.05 and Δ*β* > 0.3 were employed for screening the differential methylation distal probes. Probe–gene pairs were determined in an unsupervised manner. In the enrichment analysis of the TF motif, the 95% confidence interval (CI) of the odds ratio (OR) was more significant than 1.3 as the threshold.

### 2.8 Cell culture and siRNA transfection

Three glioma cell lines T98G, U251, and LN229 were obtained from the Procell Life Science & Technology Co., Ltd. The cells were cultured in DMEM medium (Sigma), and cells in the logarithmic growth phase were taken for subsequent experiments. Small‐interfering RNAs (siRNAs; RiboBio) and Lipofectamine 3000 (Invitrogen) were diluted with serum‐free DMEM medium, mixed, and left to stand for 20 min. The mixture was added into the well to be transfected, and after 6 h of incubation, a fresh complete culture medium was added. Cells were harvested 48 h after transfection for subsequent assays. The sequences of siRNAs are summarized in Table S2.

### 2.9 Quantitative real‐time PCR

TRIzol reagent (Invitrogen) was utilized to extract total RNA from glioma cells. The HiScript 1st Strand cDNA Synthesis Kit (Vazyme) was applied to synthesize cDNA by reverse transcription. The Taq Pro Universal SYBR qPCR Master Mix (Vazyme) was used for the quantification of lncRNA expression through quantitative real‐time PCR (qRT‐PCR). The relative Ct method was used to analyze the data, with ACTB serving as an internal reference. The primer sequences are as follows:

AGAP2‐AS1‐F: TACCTTGACCTTGCTGCTCTC.
AGAP2‐AS1‐R: TGTCCCTTAATGACCCCATCC.
ACTB‐F: CATGTACGTTGCTATCCAGGC.
ACTB‐R: CTCCTTAATGTCACGCACGAT.

### 2.10 Cell counting kit‐8 (CCK‐8) assay

Cells were digested and counted, and 2 × 10^3^ cells were seeded per well in a 96‐well plate. Using the cell counting kit‐8 (CCK‐8) kit (Genview), the absorbance value of cells at 450 nm was detected every 24 h to reflect the cell proliferation.

### 2.11 5‐Ethynyl‐2′‐deoxyuridine (EdU) assay

The 5‐ethynyl‐2′‐deoxyuridine (EdU) Cell Proliferation Assay Kit (RiboBio) was utilized to conduct EdU assays, following the instructions provided by the manufacturer. Using a 96‐well plate, cells were cultured in a complete medium containing 50 μM EdU for 4 h. After fixation and permeabilization, the cells were incubated with Apollo567 solution for 30 min, followed by staining of the nuclei with Hoechst 33342 solution. Finally, the cell plates were visualized with a fluorescence microscope.

### 2.12 Subcutaneous xenograft assay

Female BALB/c nude mice, aged 4 weeks, were acquired from the Hunan Slake Jingda Experimental Animal Co., Ltd. Well‐grown LN229 cells were prepared and transiently transfected with control and AGAP2‐AS1 knockdown siRNAs. After 24 h, the cells underwent digestion and were washed using PBS. After counting, the cells were diluted at a concentration of 5 × 10^6^ cells/100 μL. Following the administration of respiratory anesthesia to nude mice, 100 μL of control/AGAP2‐AS1 knockdown cells were injected subcutaneously. The growth of subcutaneous tumors in mice was observed. After 21 days, the mice were executed and the tumors were taken out, measured using a caliper and weighed. This animal study was approved by the Laboratory Animal Ethics Committee of The First Hospital of Hunan University of Chinese Medicine and in accordance with the guidelines for animal welfare (ZYFY20221214‐02).

### 2.13 Transwell assay

The transwell upper chambers (a pore size of 8 μm; BD Biosciences) without Matrigel were used to assess alterations in cell migration ability. After siRNA transfection for 24 h, 2 × 10^4^ cells were added to the upper chambers, and the upper chambers were cultured in a complete medium containing 20% FBS. Following 36 to 48 hours, the migrated cells were treated with 100% methanol for fixation and subsequently stained with 5% crystal violet before being counted. Matrigel was added to the bottom of the upper chambers to detect changes in cell invasion ability. Other conditions were the same as above.

### 2.14 Western Blot assay

Total cell protein was extracted with RIPA lysis buffer, and the BCA method was applied for the determination of protein concentration. Proteins were separated using SDS‐PAGE and transferred to the PVDF membrane. Then, 5% nonfat milk was added to block for 1 h. After adding the primary antibodies and incubating overnight, the secondary antibodies were added and incubated for 2 h. Finally, an ECL detection system was adopted for visualization. All antibodies were derived from Proteintech. The loading control was GAPDH.

### 2.15 Statistical analysis

Statistical analysis of this study was performed using R software (version 3.5.3). The Shapiro–Wilk test was used to assess whether the data conform to a normal distribution. Comparisons of normally distributed variables between two groups and multiple groups were implemented by Student's *t*‐test and one‐way analysis of variance (ANOVA), respectively. The Wilcoxon test and the Kruskal–Wallis test were applied to compare the non‐normally distributed data between the two groups and multiple groups, respectively. Survival analysis was carried out with the “survival” R package, using the Kaplan–Meier method and log‐rank test. *p* < 0.05 was employed as the threshold to determine a statistically significant distinction.

## 3 RESULTS

### 3.1 A prognostic TSRlncRNA signature was established

**FIGURE 1:**
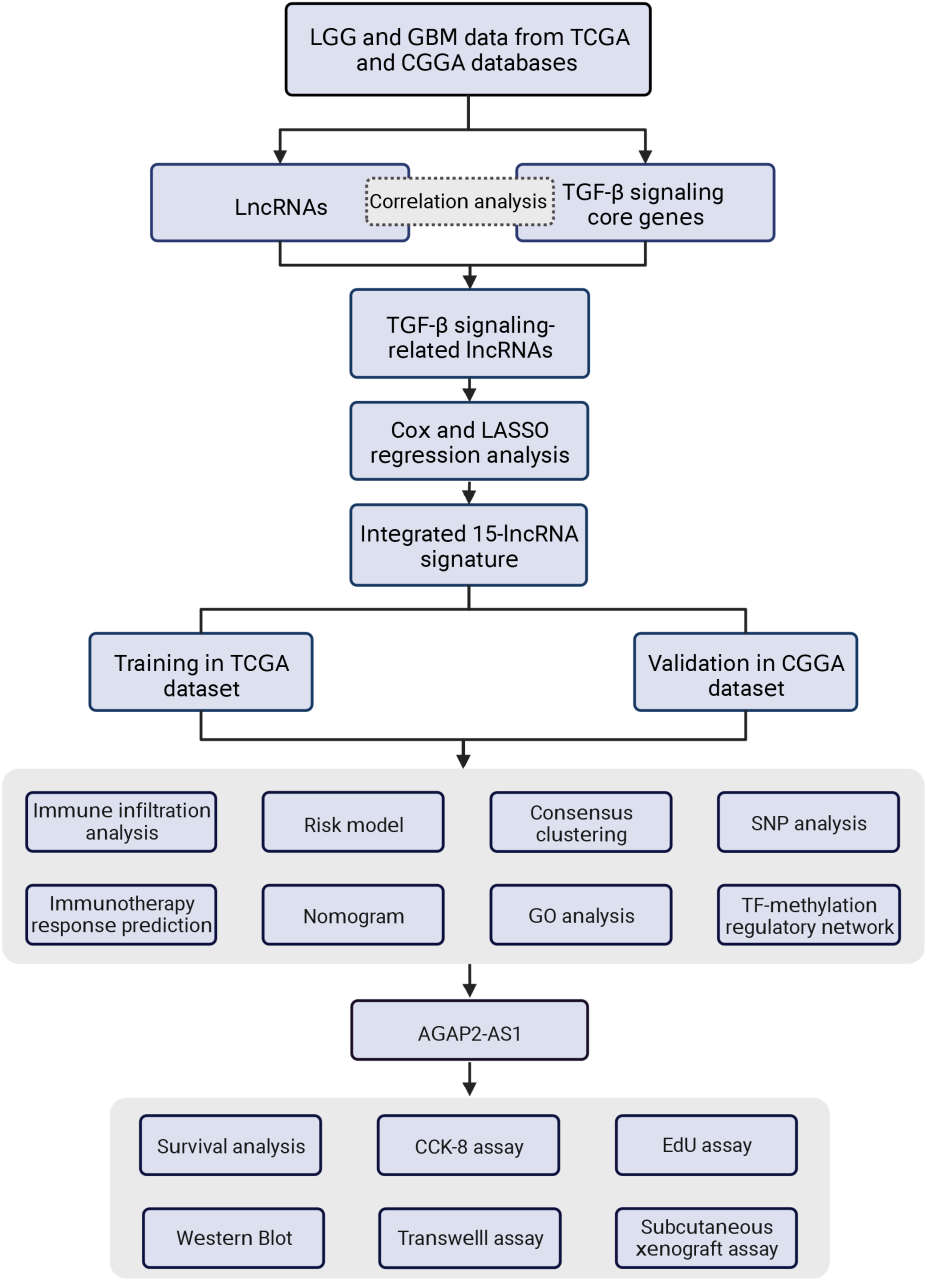
The flowchart of this study.

**FIGURE 2:**
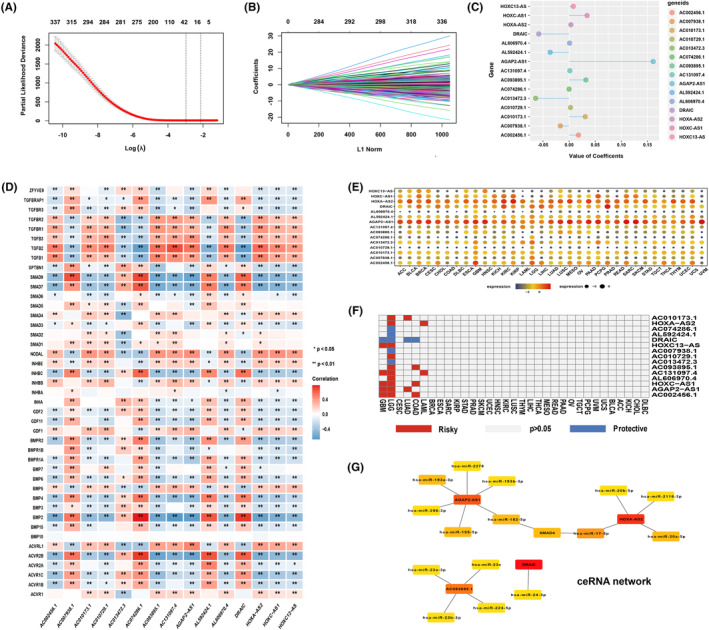
(A) The ten‐time cross‐validation plot of LASSO regression. (B) LASSO coefficient plot for lncRNAs with independent prognostic value. (C) LASSO coefficient profile of the 15 signature lncRNAs. (D) Correlation between signature lncRNAs and TGF‐β signaling core genes. (E) Expression levels of signature lncRNAs in the pan‐cancer cohort. (F) The impact of signature lncRNAs on pan‐cancer prognosis. (G) The ceRNA network based on signature lncRNAs.

The study flow chart is demonstrated in Figure 1. The 43 TGF‐β signaling core genes (TSCGs) are listed in Table S3. We uncovered 1142 TSRlncRNAs by the correlation analysis between TSCG expression and lncRNA expression. The above TSRlncRNAs were incorporated in Cox regression analysis, and 382 survival‐related lncRNAs were screened out. Finally, we obtained 15 TSRlncRNAs with the most prognostic value through LASSO regression analysis, including AC010173.1, HOXA‐AS2, AC074286.1, AL592424.1, DRAIC, HOXC13‐AS, AC007938.1, AC010729.1, AC013472.3, AC093895.1, AC131097.4, AL606970.4, HOXC‐AS1, AGAP2‐AS1, and AC002456.1 (Figure 2A–C). Hence, a lncRNA signature of TGF‐β signaling was established in glioma. Figure 2C shows the LASSO regression coefficients of each signature TSRlncRNA. The correlation between the signature lncRNAs and TGF‐β signaling is displayed in Figure 2D.

In the TCGA cohort, patients were assigned to high and low expression groups based on the median expression value of each signature TSRlncRNA. We compared the prognosis (overall survival, OS) of the two groups and observed notable disparities (*p* < 0.001, Figure S1). The high expression of AC010173.1, HOXC‐AS1, HOXA‐AS2, HOXC13‐AS, AC093895.1, AGAP2‐AS1, AC131097.4, AC002456.1, AC010729.1, and AL606970.4 suggested poor prognosis, indicating their role as risk lncRNAs in glioma, while AC074286.1, AL592424.1, DRAIC, AC007938.1, and AC013472.3 were protective lncRNAs. In the CGGA cohort, we also obtained mostly similar results, where their high or low expression significantly affected glioma survival (*p* < 0.001, Figure S2).

We further performed a pan‐cancer analysis. The expression of signature lncRNAs in the pan‐cancer cohort is demonstrated in Figure2E. We assessed the prognostic significance of signature TSRlncRNAs in pan‐cancer and found that they predominantly affected the OS of glioma (Figure 2F), suggesting the ralatively specificity of the signature lncRNAs we screened in glioma. In addition, we also constructed a ceRNA network of TGF‐β signaling (Figure 2G). We found that SMAD4 was the hub gene in the network and identified two novel regulatory axes of TGF signaling, AGAP2‐AS1/miR‐182‐5p/SMAD4, and HOXA‐AS2/miR‐17‐5p/SMAD4.

### 3.2 Two novel glioma TSRlncRNA subtypes were identified

**FIGURE 3:**
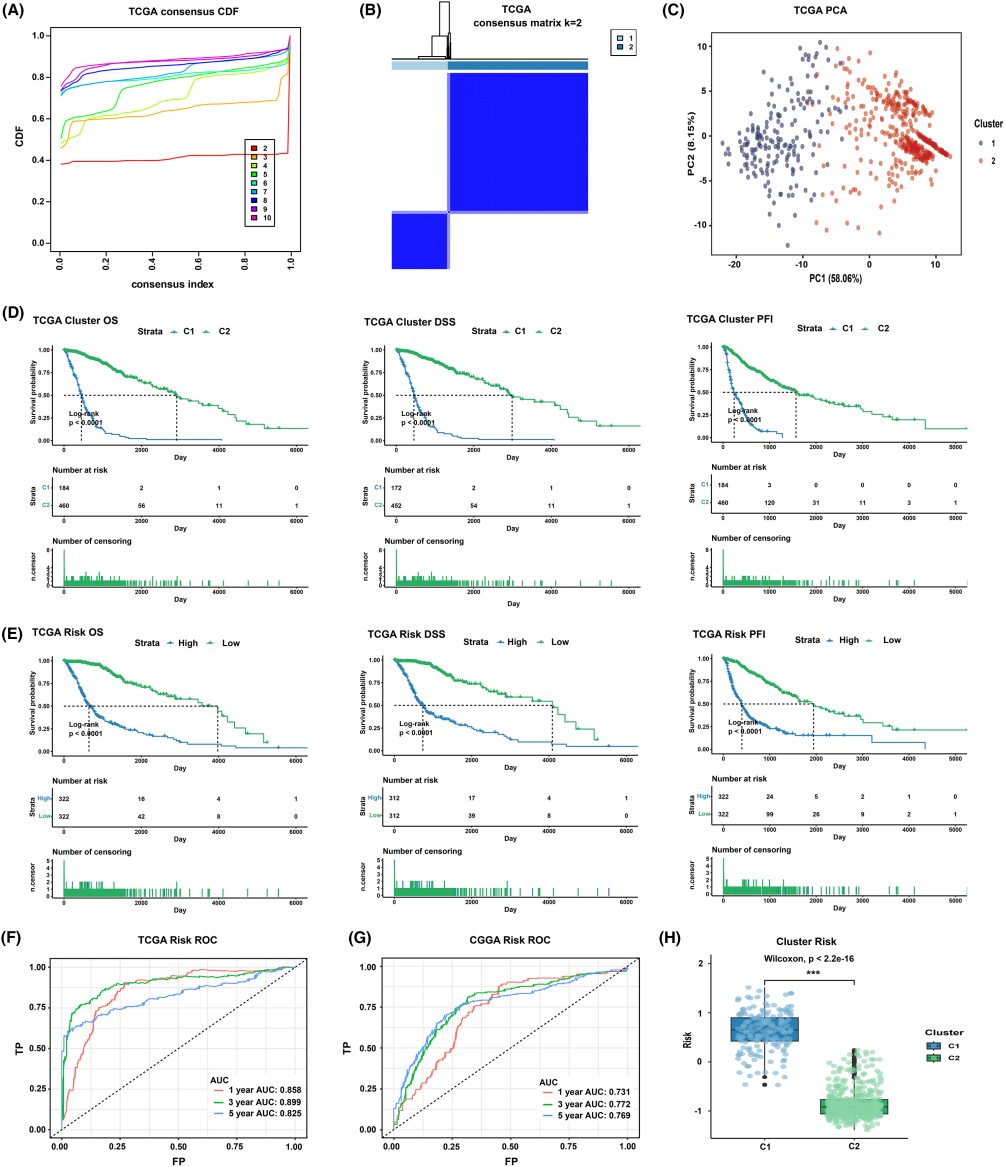
(A) The CDF curves of k value from 2 to 10. (B) Heatmap of consensus matrix when *k* = 2. (C) Principal component analysis. (D) Prognostic comparison between cluster 1 and cluster 2. (E) Comparison of clinical outcomes between high‐ and low‐risk groups. (F) 1‐, 3‐, and 5‐year ROC curves and AUC values of the risk model in the TCGA dataset. (G) 1‐, 3‐, and 5‐year ROC curves and AUC values of the risk model in the CGGA dataset. (H) Comparison of risk scores for cluster 1 and cluster 2.

By consensus clustering based on the signature TSRlncRNA expression profile, we identified two robust glioma TSRlncRNA subtypes in the TCGA cohort, cluster 1 and cluster 2 (Figure 3A,B). In PCA, the two subtypes had a good degree of discrimination, which supported the clustering results (Figure 3C). We compared the prognosis of the two subtypes. The findings indicated that cluster 2 exhibited superior OS, disease‐specific survival (DSS), and progression‐free interval (PFI) compared to cluster 1 in the pan‐glioma cohort (*p* < 0.0001, Figure 3D). In LGG and GBM cohorts, cluster 2 also demonstrated a more favorable prognosis (*p* < 0.01, Figure S3A,B). Furthermore, we validated the clustering analysis results using the CGGA dataset. In the CGGA pan‐glioma, LGG, and GBM cohorts, we similarly obtained two subtypes with distinct prognoses, with cluster 2 having a significantly better prognosis (*p* < 0.05, Figure S3C,D).

### 3.3 TSRlncRNA signature could effectively distinguish patients with different prognoses

Based on the TSRlncRNA signature, we constructed a risk scoring model. The associations between risk score and TSCGs as well as signature lncRNAs are shown in Figure S4. We calculated patients' risk scores and stratified them into high‐ and low‐risk groups based on the median obtained. Survival analysis indicated a notable association between high‐risk and unfavorable prognoses. In the pan‐glioma cohort, the prognosis of high‐risk patients was considerably poorer compared to low‐risk patients regardless of which prognostic indicator was used (OS, DSS, and PFI) (*p* < 0.0001, Figure 3E). Comparable findings were observed in TCGA GBM and LGG cohorts (*p* < 0.01, Figure S5A,B). We further validated the results using the CGGA pan‐glioma, LGG, and GBM cohorts and also found that high risk was indicative of a poor prognosis (*p* < 0.05, Figure S5C). We further calculated the AUC values of the risk model. In the TCGA dataset, the AUC values of the 1‐, 3‐, and 5‐year ROC curves were 0.858, 0.899, and 0.825, respectively (Figure 3F), and the corresponding AUC values were 0.731, 0.772, and 0.769 in the CGGA dataset (Figure 3G). In addition, we also evaluated the consistency of the clustering results with the risk model and observed that a high‐risk score appeared in cluster 1 with poor prognosis (*p* < 0.001, Figure 3H). Hence, we believed that the risk‐scoring model of TSRlncRNA had an excellent ability to predict the glioma prognosis.

### 3.4 TSRlncRNA signature had an independent prognostic value

To determine the independence of this TSRlncRNA signature in prognostic assessment, we further conducted Cox regression analysis. Risk score and clinicopathological characteristics including age, WHO grade, IDH mutation status, 1p/19q codeletion status, and subtype (classical, mesenchymal, neural, and proneural) were incorporated in this analysis. In univariate Cox regression analysis, the risk score was identified as a survival‐related factor (HR 4.66, *p* < 0.001, Table S4). Furthermore, multivariate Cox regression analysis showed that the risk score possessed an independent prognostic value (HR 2.88, *p* < 0.001, Table S5).

**FIGURE 4:**
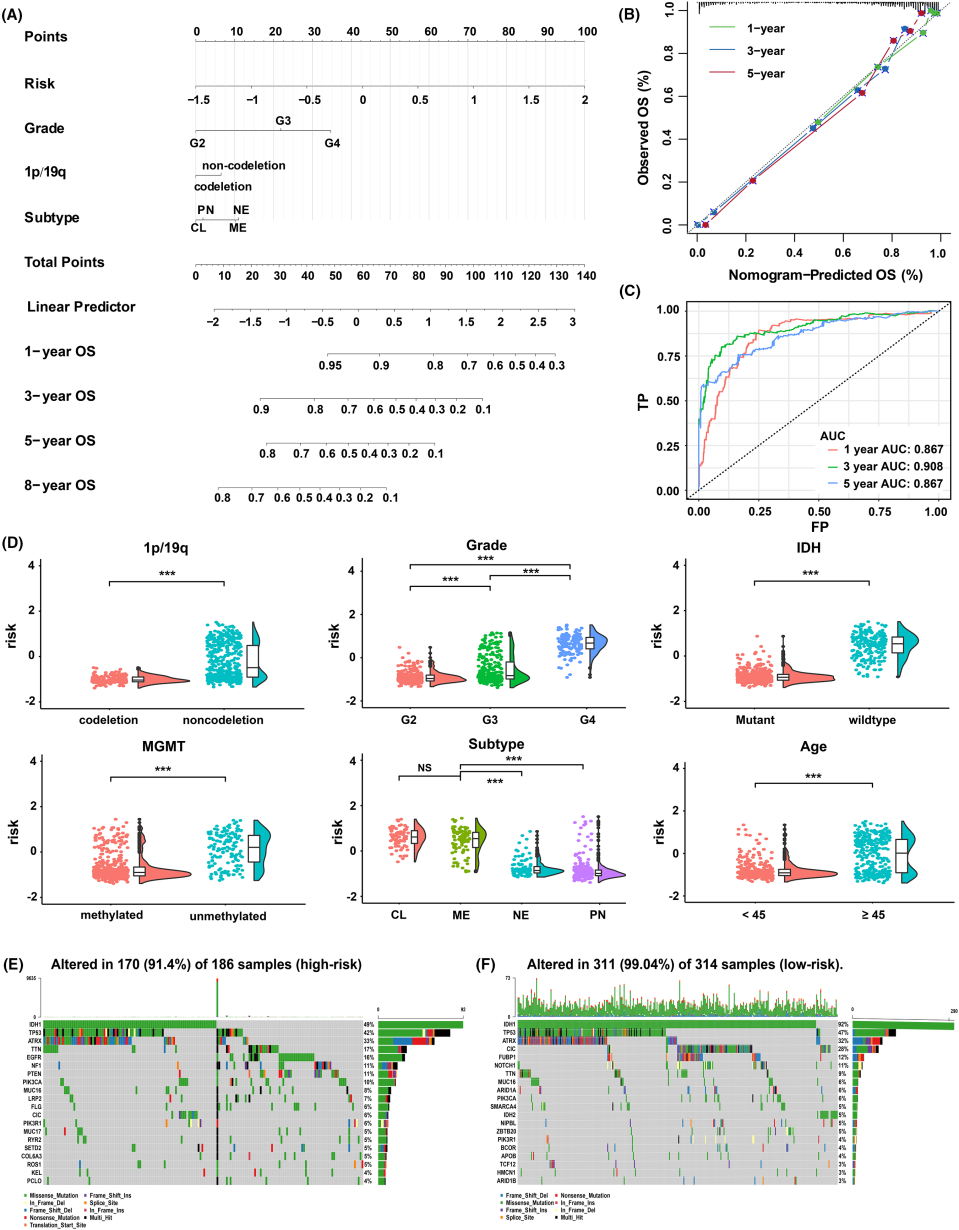
(A) Nomogram was used to predict OS by adding up the points of each variable. The total points projected on the bottom scale represent the predicted survival probability. (B) The bootstrapping calibration curves for the nomogram. The x‐axis and y‐axis are predicted OS and actual OS, respectively. The gray dashed line corresponds to perfect prediction. (C) 1‐, 3‐, and 5‐year ROC curves and AUC values of the nomogram. (D) Subgroup analysis of risk score. (E, F) The somatic mutation profiles in high‐risk (E) and low‐risk (F) groups. OS, overall survival; NS, no significance, ***p < 0.001.

To enhance clinical utility, we further developed a nomogram by integrating the clinicopathological characteristics and risk score (Figure 4A). In the nomogram, each factor was assigned a number of weighted points, and the points for each factor of the patient were added up, and the sum corresponds to the predicted OS. As shown in the bootstrapped calibration plot, the nomogram exhibited good reliability with calibration curves all approaching the 45‐degree line (Figure 4B). Patients were classified into high‐ and low‐point groups based on the median of the points obtained. Survival analysis indicated that the high‐point group had a poorer prognosis compared to the low‐point group (*p* < 0.0001, Figure S6A). The nomogram AUC values were 0.867 (1‐year OS), 0.908 (3‐year OS), and 0.867 (5‐year OS), all better than those of the risk model (Figure 4C). The nomogram showed satisfactory discrimination strength in prognosis.

### 3.5 TSRlncRNA signature correlated with clinicopathological and genomic mutation characteristics

To comprehend the correlation between the risk score and the clinicopathological characteristics, a subgroup analysis was conducted. We divided patients into different subgroups: age (≥45, <45), gender (male, female), IDH (wild‐type, mutant), 1p/19q (codeletion, non‐codeletion), WHO grade (G2, G3, and G4), MGMT (unmethylated, methylated), and subtype (classical, mesenchymal, neural, and proneural). By comparing risk scores between subgroups, it was observed that patients with age ≥45, high WHO grade, IDH‐wild‐type, MGMT promotor unmethylated, 1p/19q non‐codeletion, and classical and mesenchymal subtypes had a higher preference for a high‐risk score distribution (*p* < 0.001, Figure 4D). In the gender subgroup, males and females did not differ in risk scores (*p* > 0.05, Figure S6B).

The somatic mutation status of groups at high and low risks was examined. Somatic mutations were detected in 91.4% of samples with high risk and 99.04% of samples with low risk (Figure 4E,F). By comparing the mutational profiles of the two groups, we observed that high‐risk patients had higher mutation frequencies in EGFR, PTEN, NF1, ROS1, SETD2, and LRP2 genes, whereas low‐risk patients had more frequent mutations in IDH1, CIC, NOTCH1, FUBP1, and SMARCA4 (*p* < 0.001, Figure S6C). These findings indicate that patients at high and low risks exhibit distinct clinicopathological and genomic mutation features.

### 3.6 TSRlncRNA signature suggested immune infiltration characteristics

**FIGURE 5:**
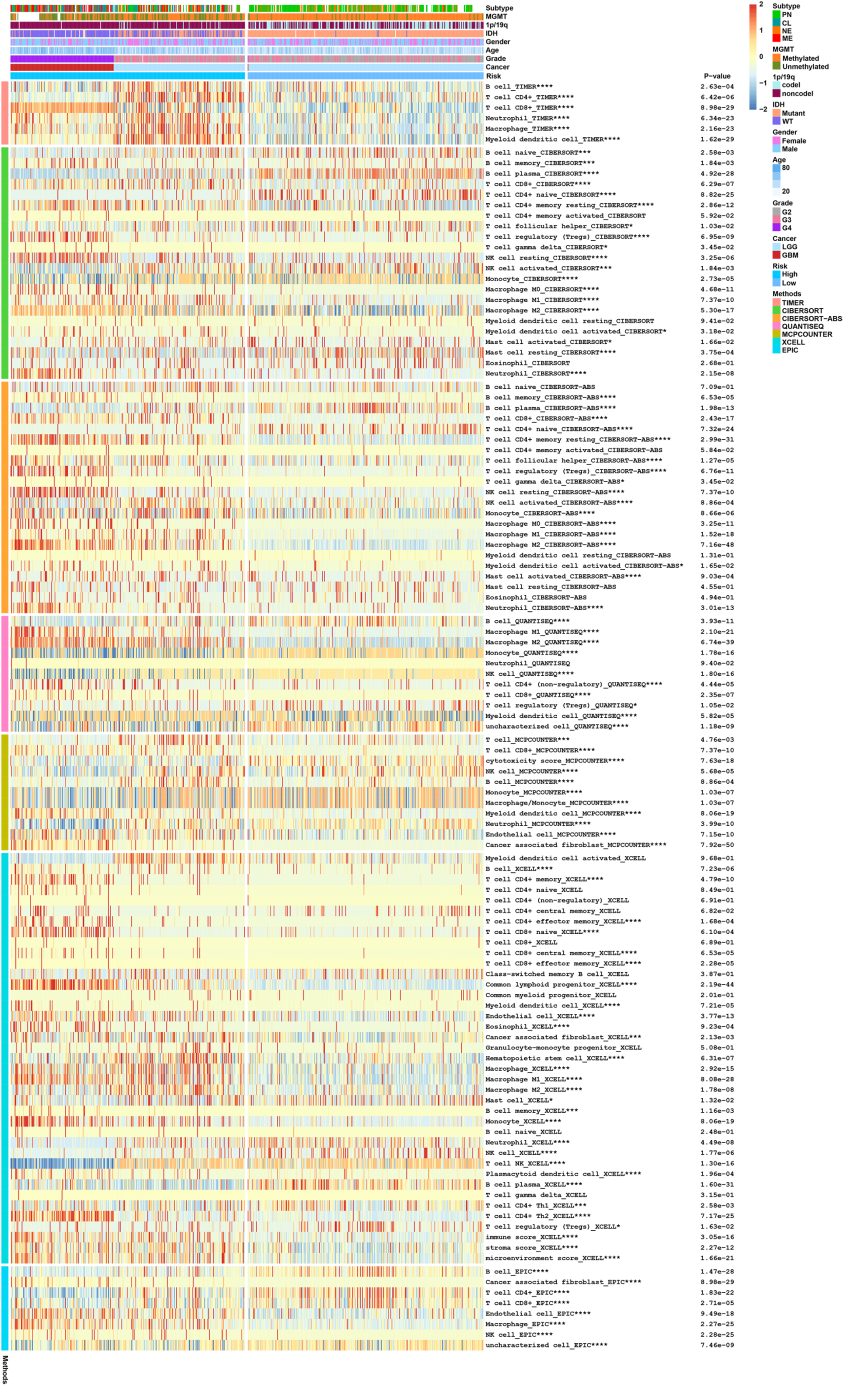
Analysis of immune infiltration characteristics of high‐ and low‐risk patients using TIMER2.0 database.

TGF‐β signal is an essential regulator of immune activities, and the tumor immune microenvironment (TIME) significantly influences tumor development, metastasis, and outcomes.^32^ Thus, we investigated the correlation between the TSRlncRNA signature and the immune microenvironment in glioma. By utilizing multiple algorithms from the TIMER2.0 database, we assessed the immune traits of patients categorized as high and low risks. Our analysis revealed notable disparities in the immune infiltration patterns between the two groups. In high‐risk patients, there was a notable increase in the level of macrophage infiltration (Figure 5). Furthermore, the GO analysis indicated a notable enrichment of immune‐related biological processes, such as neutrophil activation involved in immune response, T‐cell activation, immune response‐activating signal transduction, and lymphocyte‐mediated immunity (Figure S6D). The above results revealed the essential biological significance of signature TSRlncRNAs in the glioma immune microenvironment.

### 3.7 TSRlncRNA signature indicated immunotherapeutic response

**FIGURE 6:**
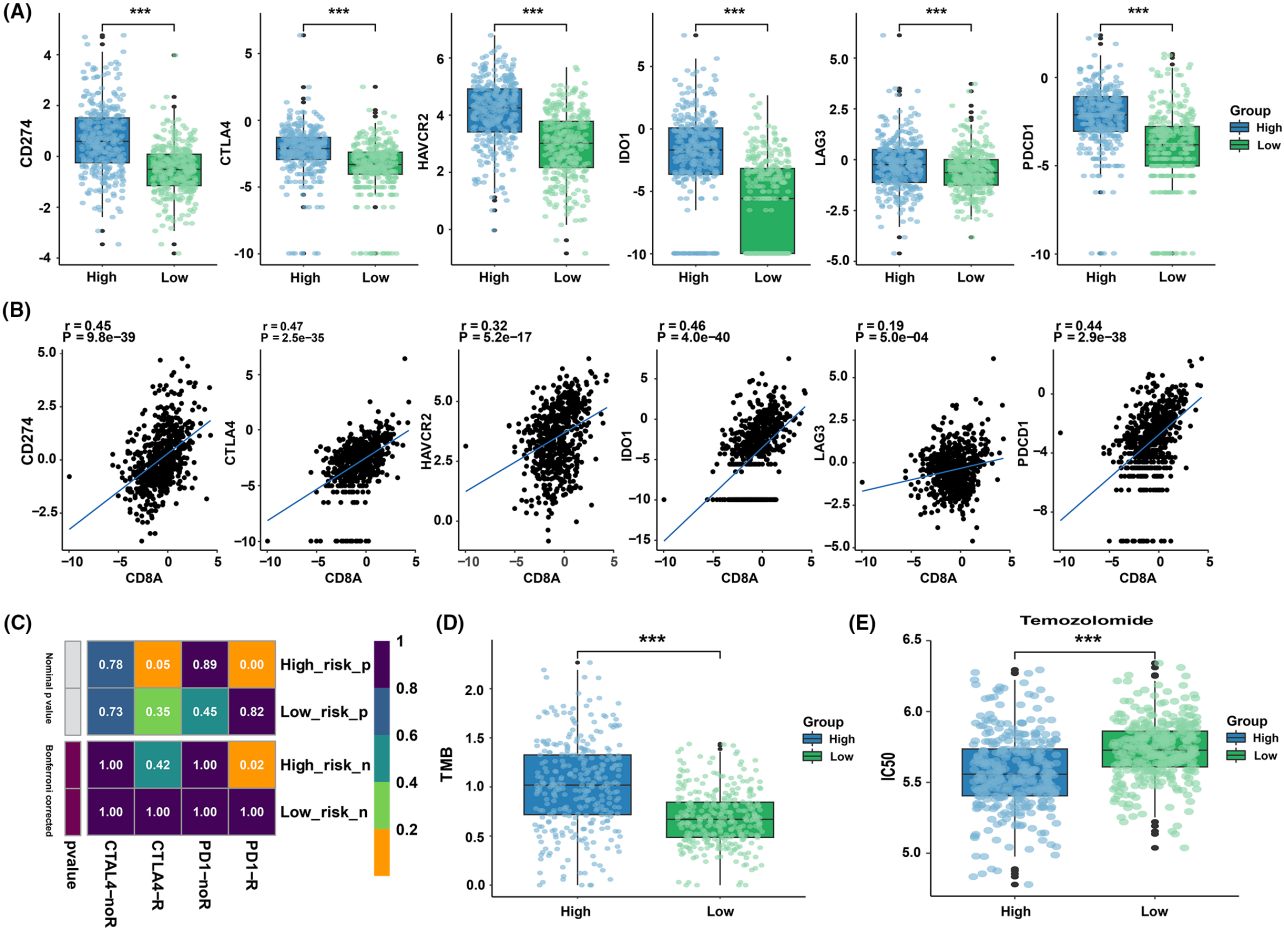
(A) High‐risk patients showed higher expression levels of immune checkpoint molecules. (B) Correlation between CD8A expression and immune checkpoint expression. (C) The sensitivity analysis of anti‐PD‐1 and anti‐CTLA4 treatment in high‐ and low‐risk groups. (D) Tumor mutational burden analysis. (E) Comparison of temozolomide treatment sensitivity between high‐ and low‐risk groups. ***p < 0.001.

We further analyzed the expression of immune checkpoint molecules in samples and observed that CD274 (PD‐L1), CTL4, HAVCR2 (TIM‐3), IDO1, LAG3, and PDCD1 (PD‐1) were highly expressed in high‐risk patients (*p* < 0.001, Figure 6A). The upregulation of tumor cell adaptive immune resistance pathways is related to the production of interferon‐gamma (IFN‐γ) by CD8+ T cells in TIME.^33^ Hence, we also investigated the relationship between CD8+ T cell marker CD8A and immune checkpoints in glioma. The findings showed a strong positive correlation between their expressions (*p* < 0.001, Figure 6B), which is in line with previous study reported.^34^ Moreover, we predicted the immunotherapy response. The results indicated that high‐risk suggested better anti‐PD‐1 treatment responsiveness (*p* = 0.02, Figure 6C). No differences in sensitivity to anti‐CTLA4 therapy were observed between patients at high and low risks (*p* > 0.05, Figure 6C). Moreover, patients at a high risk displayed a higher tumor mutation burden (TMB) (*p* < 0.001, Figure 6D). In chemosensitivity prediction, high‐risk patients had better chemotherapy responsiveness, as indicated by a significantly lower estimated IC_50_ value for temozolomide (TMZ) (*p* < 0.001, Figure 6E).

### 3.8 TF‐methylation‐gene regulatory network associated with signature TSRlncRNAs

**FIGURE 7:**
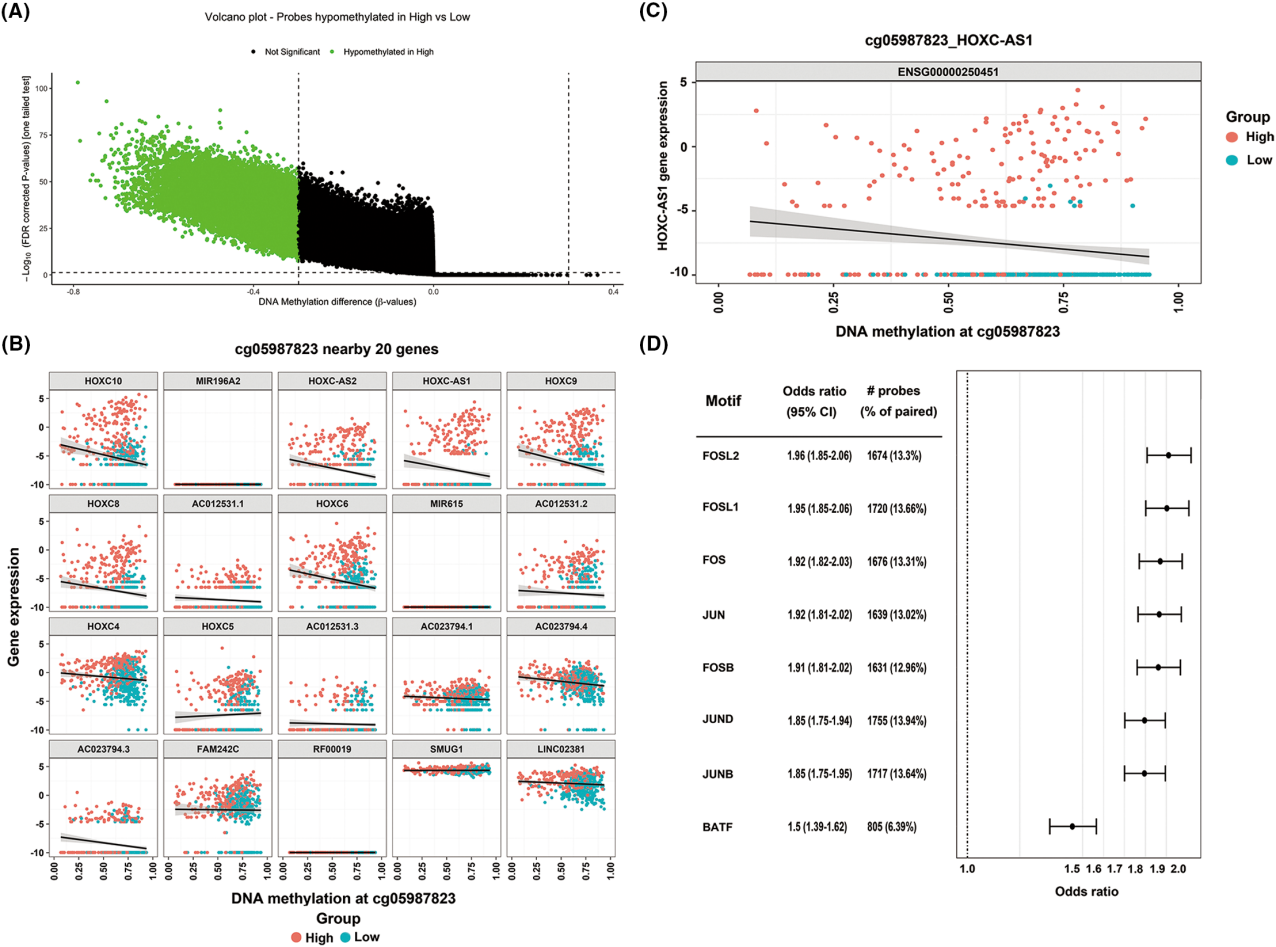
(A) Volcano plot showing differentially hypomethylated distal probes between high‐ and low‐risk patients. (B) Scatter plots showed the correlation between the methylation level of probe cg05987823 and the expression of 20 adjacent genes. (C) Correlation analysis of the HOXC‐AS1 expression and the methylation level of probe cg05987823. (D) Motif enrichment plot showed the enrichment levels of the most significant motifs based on unsupervised analysis.

In order to gain a deeper comprehension of the regulatory mechanism behind the expression of signature lncRNAs in glioma, we explored the cis‐regulatory interface of the gene transcription of signature lncRNAs. By comparing the methylation levels of distal probes in high‐ and low‐risk patients (Figure 7A), we identified cg05987823 as the most differentially hypomethylated site. To understand which genes' transcription is affected by this methylation site, the correlation between the expression levels of 10 upstream genes and 10 downstream genes closest to the site and the methylation level at this site was evaluated. As shown in Figure 7B, several probe–gene pairs were identified, the signature lncRNA HOXC‐AS1 was included, and its expression level exhibited an inverse correlation with the cg05987823 methylation level (Figure 7C, enlarged from HOXC‐AS1 in Figure 7B). We performed motif enrichment analysis of the differentially methylated distal probes further to determine the upstream TFs. The results indicated that the TF binding site motifs for FOSL2, FOSL1, FOS, JUN, FOSB, JUND, JUNB, and BATF were significantly over‐represented (Figure 7D). The above results reveal a TF‐methylation‐gene regulatory network associated with signature TSRlncRNAs and glioma progression.

### 3.9 AGAP2‐AS1 correlated with glioma prognosis, immune infiltration status, and treatment response

**FIGURE 8:**
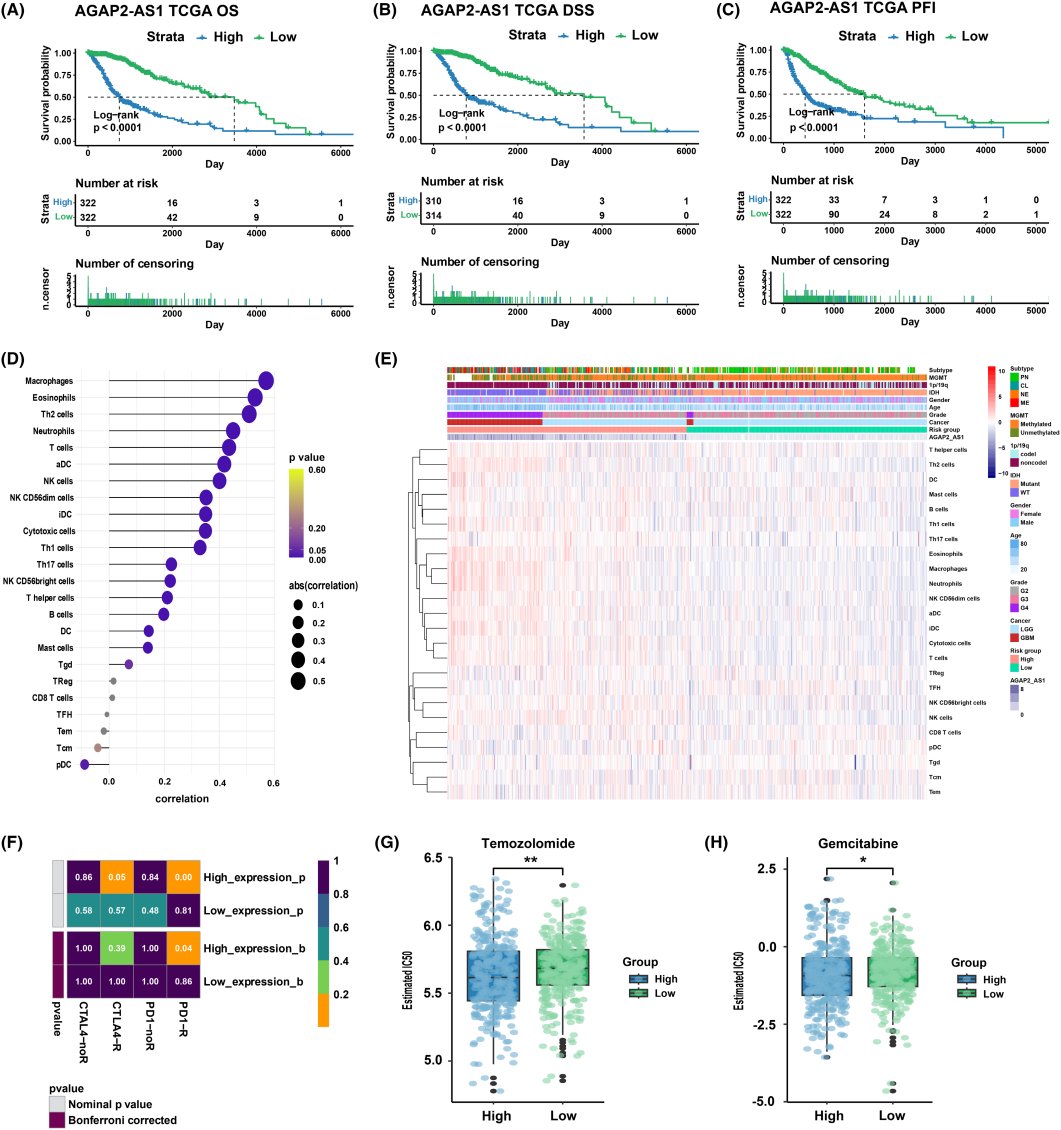
(A–C) Differences in prognosis between high and low AGAP2‐AS1 patients. (D) Correlation analysis between AGAP2‐AS1 expression and immune cell infiltration. (E) Heatmap showed the infiltration levels of immune cells in high and low AGAP2‐AS1 groups. (F) The sensitivity analysis of anti‐PD1 and anti‐CTLA4 treatment in high and low AGAP2‐AS1 groups. (G, H) The estimated IC_50_ values of temozolomide (G) and gemcitabine (H) in high and low AGAP2‐AS1 groups. *p < 0.05, **p < 0.01.

As the risk gene with the most significant weight in the risk model, we selected AGAP2‐AS1 for further analysis. We used OS, DSS, and PFI as outcome indicators to comprehensively assess its impact on prognosis. The findings indicated that a correlation existed between the high expression of AGAP2‐AS1 and unfavorable prognosis (*p* < 0.0001, Figure 8A–C). We also explored the association between TIME and AGAP2‐AS1, and observed a positive correlation between AGAP2‐AS1 expression and the macrophage infiltration (*p* < 0.05, Figure 8D,E). Meanwhile, treatment sensitivity was assessed. Patients with high AGAP2‐AS1 responded better to anti‐PD‐1 therapy and chemotherapy (*p* < 0.05, Figure 8F–H).

### 3.10 Tumor suppressor effect of AGAP2‐AS1 knockdown

**FIGURE 9:**
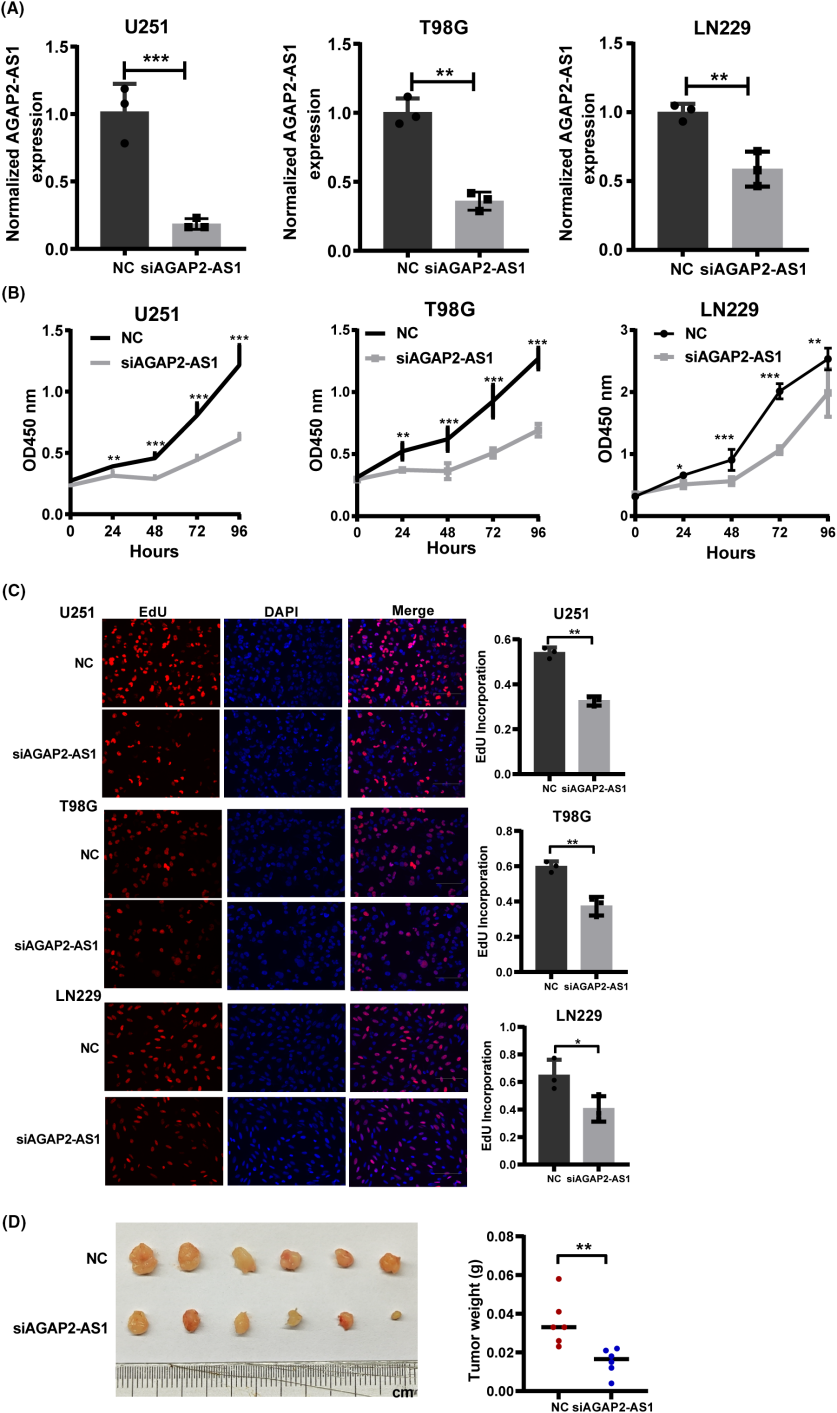
(A) The AGAP2‐AS1 expression in the negative control (NC) and siRNA‐AGAP2‐AS1 (siAGAP2‐AS1) groups was detected using qRT‐PCR. (B) In CCK‐8 assays, cell proliferative activity was assessed every 24 h after silencing the expression of AGAP2‐AS1. (C) In EdU assays, the knockdown of AGAP2‐AS1 inhibited the proliferation ability of glioma cells. (D) In subcutaneous xenograft assay, AGAP2‐AS1 knockdown inhibited glioma growth. *p < 0.05, **p < 0.01, ***p < 0.001.

**FIGURE 10:**
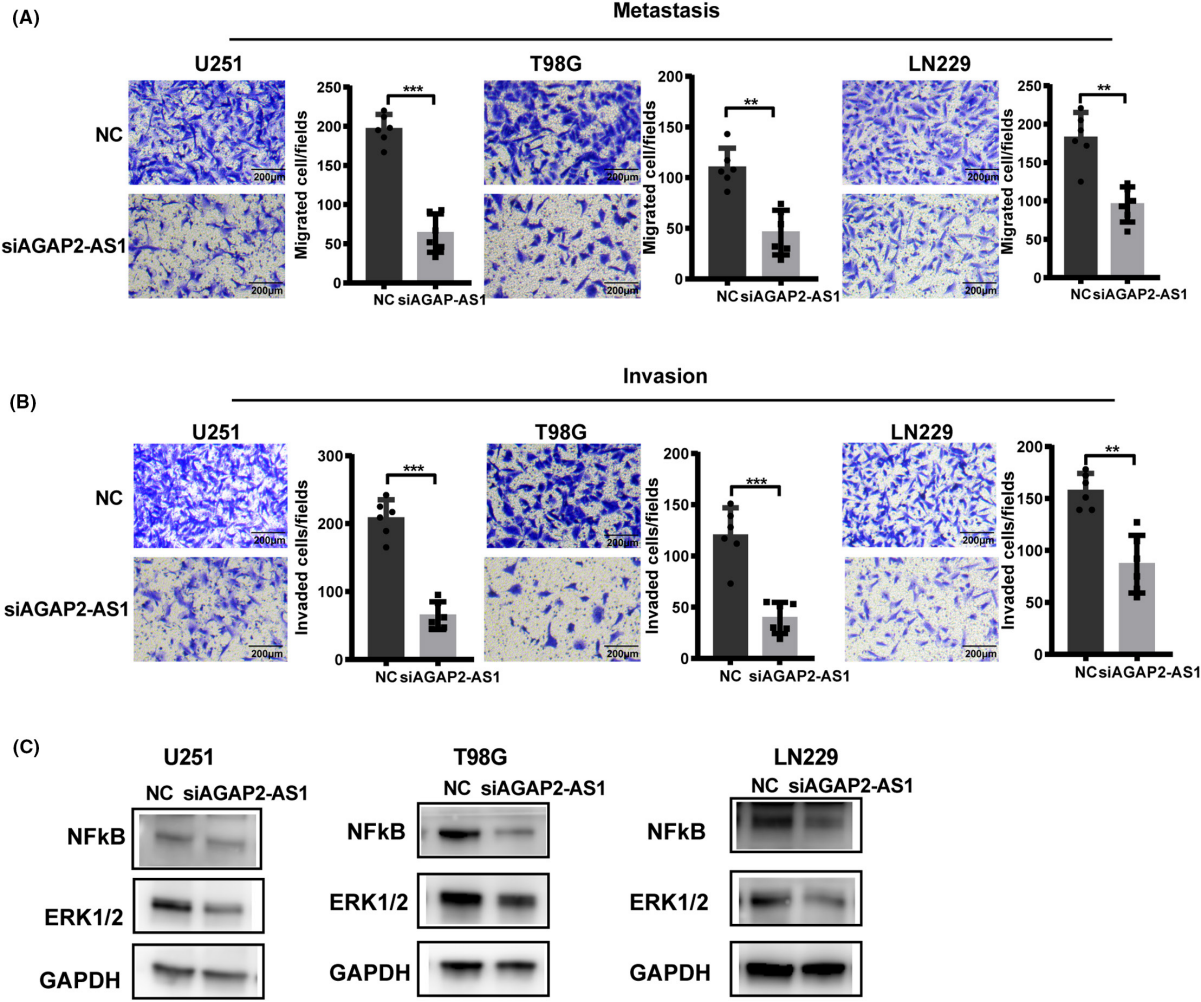
(A) In transwell assays without Matrigel, the knockdown of AGAP2‐AS1 inhibited the migration capacity of glioma cells; scale bar: 200 μm. (B) In Matrigel invasion assays, AGAP2‐AS1 knockdown inhibited the invasive capacity of glioma cells; scale bar: 200 μm. (C) In western blot assays, AGAP2‐AS1 knockout significantly reduced NF‐κB and ERK1/2 expression. **p < 0.01, ***p < 0.001.

We conducted in vitro experiments utilizing three glioma cell lines U251, T98G, and LN229. The impact of AGAP2‐AS1 on the glioma's biological behavior was examined by using siRNA to silence AGAP2‐AS1. With the control group as a reference, the siRNA group exhibited a significant decrease in AGAP2‐AS1 expression, suggesting the effective silencing of AGAP2‐AS1 by siRNA (Figure 9A). The results of CCK‐8 assays showed that the proliferation viability of glioma cells was suppressed after silencing AGAP2‐AS1 expression (Figure 9B). The EdU assays also indicated that AGAP2‐AS1 silencing impaired the proliferation of glioma cells (Figure 9C). In in vivo experiments, AGAP2‐AS1 knockdown led to a notable reduction in both the volume and weight of xenograft tumors when compared to the control group (Figure 9D). We further evaluated the impact of AGAP2‐AS1 on the migration and invasion of glioma cells. The results of transwell assays suggested that AGAP2‐AS1 silencing markedly restrained the metastasis and invasion of glioma cells (Figure 10A,B). These findings suggested that AGAP2‐AS1 knockdown suppressed the proliferation, migration, and invasion of glioma cells in vitro, as well as hindered the growth of glioma in vivo.

We further explored the possible mechanism of AGAP2‐AS1 affecting glioma. Western blot analysis revealed that knockdown of AGAP2‐AS1 resulted in a significant decrease in nuclear factor‐kappaB (NF‐κB) and extracellular signal‐regulated kinase (ERK) 1/2 protein levels in glioma cells (Figure 10C). The results of in vitro and in vivo experiments confirmed the important role of AGAP2‐AS1 in glioma development, and its regulation in glioma may be realized by downregulating the expression of NF‐κB and ERK 1/2 in the TGF‐β signaling pathway.

## 4 DISCUSSION

In this research, we established a TSRlncRNA signature in glioma and 15 TSRlncRNAs that possess prognostic significance were identified, including AC010173.1, HOXA‐AS2, AC074286.1, AL592424.1, DRAIC, HOXC13‐AS, AC007938.1, AC010729.1, AC013472.3, AC093895.1, AC131097.4, AL606970.4, HOXC‐AS1, AGAP2‐AS1, and AC002456.1. Among them, AC093895.1, AC007938.1, AC010173.1, AC010729.1, AL592424.1, and AL606970.4 have not yet been reported on functional studies. AC002456.1 was identified as a high‐risk immune‐related lncRNA in GBM.^35^ In lipopolysaccharide‐stimulated alveolar macrophages, AC013472.3 may suppress the secretion of tumor necrosis factor‐alpha by interfering with the NF‐κB signaling pathway.^36^ Its role in tumors has not been reported yet. In this study, it was significantly associated with glioma survival, but for the positive and negative associations, we got conflicting results in the TCGA and CGGA cohorts. This result may have been confounded by inconsistencies in ethnicity and sample size. Given the notably larger sample size of the TCGA cohort, we consider AC013472.3 to be a protective lncRNA in glioma. AC074286.1 is involved in epigenetic remodeling of nonalcoholic fatty liver disease.^37^ AGAP2‐AS1 facilitates the occurrence and progression of certain tumors, including intrahepatic cholangiocarcinoma, breast cancer, and lung cancer, and its upregulation is linked to a poor prognosis.^38^, ^39^, ^40^ HOXA‐AS2 is upregulated in cervical cancer, acute myeloid leukemia, and hepatoblastoma, promoting malignant biological behavior.^41^, ^42^, ^43^ AC131097.4 is related to genomic instability of LGG, and its high expression suggests the possibility of a wild‐type IDH1 gene.^44^ DRAIC is a suppressor in gastric and colorectal cancer cells.^45^, ^46^ HOXC‐AS1 promotes gastric cancer growth and metastasis in vivo and in vitro,^47^ and its silence hinders the growth of castration‐resistant prostate cancer.^48^ HOXC13‐AS is a tumor facilitator in head and neck squamous cell carcinoma^49^ and oral squamous cell carcinoma.^50^ The results of the above studies are similar to ours.

We identified two novel TGF‐β signaling regulatory axes, AGAP2‐AS1/miR‐182‐5p/SMAD4 and HOXA‐AS2/miR‐17‐5p/SMAD4, with SMAD4 acting as the hub gene in the ceRNA network. SMAD4 is the central mediator of TGF‐β signaling, and its function and signaling pathway abnormalities significantly contribute to the cancer genesis and progression.^51^ Additionally, we found that the expression of signature lncRNAs in glioma may be regulated by the activator protein‐1 (AP‐1) TF family, which consists of several sub‐families: the activating transcription factor (BATF, LRF1/ATF3, JDP1, JDP2, ATF2), FOS (FOS, FOSB, FOSL1, and FOSL2), and JUN (JUN, JUNB, and JUND).^52^, ^53^ AP‐1 TFs are thought to be cancer drivers, and their dysregulation is associated with tumorigenesis. Elevated AP‐1 activity has been detected in a variety of cancers, where its high expression in invasive cancers mediates proliferation and migration.^52^, ^54^, ^55^

Based on the TSRlncRNA signature, we obtained two TSRlncRNA glioma subtypes with significant differences in prognosis. A risk scoring model was further constructed, and a correlation was found between high‐risk and unfavorable prognosis. The excellent AUC values guaranteed the accuracy of the model. A good agreement was found between the risk score and the malignant clinicopathological and genomic aberration characteristics. The high WHO grade, 1p/19q non‐codeletion, MGMT promotor unmethylated, classical and mesenchymal subtypes, and IDH‐wild type, which are associated with adverse outcomes,^56^, ^57^ had high‐risk scores. Benign mutations IDH1, CIC, NOTCH1, and FUBP1^58^ had a higher prevalence in low‐risk patients, whereas malignant driving mutations EGFR, PTEN, NF1, SETD2, and LRP2^59^, ^60^, ^61^ showed a greater occurrence in high‐risk patients. Moreover, the risk score had the potential to function as a standalone prognostic indicator. These results indicated that this TSRlncRNA signature can be trusted for predicting the prognosis of glioma. The nomogram's further construction enhances the TSRlncRNA signature's clinical practicability.

TGF‐β signaling is a pivotal enforcer of immune tolerance and homeostasis, inhibiting the expansion and function of the immune system. Perturbations in TGF‐β signaling are central within the tumor immunosuppressive microenvironment and promote tumorigenesis.^62^ As a lncRNA signature of TGF‐β signaling, it can be utilized to distinguish the different immune infiltration status of glioma patients. High risk suggested high levels of macrophage infiltration, which shapes glioma immune microenvironment, suppresses the anti‐tumoral immune response, and supports tumor angiogenesis, invasion, and proliferation.^63^, ^64^, ^65^ Furthermore, a correlation was observed between high risk and increased expression of immune checkpoint molecules. Functional analysis also showed that signature lncRNAs were involved in regulating immune‐related biological processes and pathways. These results suggest that signature TSRlncRNAs are essential participants in glioma immune evasion.

Immune checkpoint blockade is a promising approach to activating therapeutic anti‐tumor immunity.^66^ However, it has a specific selectivity for the patient group that will benefit.^67^ Given the close relationship between the TSRlncRNA signature and glioma immune microenvironment, we also wonder whether it can serve as a biomarker for predicting immunotherapy response. The findings indicated that patients at a high risk showed better sensitivity to anti‐PD1 therapy. A high expression of PD1/PD‐L1 and a high TMB in high‐risk patients also suggest a better response rate to immunotherapy.^68^, ^69^

AGAP2‐AS1, which carries the most enormous weight in the risk model, was selected for further analysis. AGAP2‐AS1, an antisense lncRNA of 1567 nucleotides in length and located at 12q14.1, its upregulation is linked to unfavorable prognosis in some cancers.^70^ In the TCGA cohort of this study, its high expression suggested a poor prognosis in glioma, which was inconsistent with the results in the CGGA cohort. However, based on its prevalence as an oncogene in previous bioinformatics analysis related‐literature reports,^71^, ^72^ combined with the results validated in our subsequent experiments, AGAP2‐AS1 is considered to be a risk lncRNA for glioma, and the result of CGGA is considered to be confounded by its small sample size. Furthermore, it affected the immune microenvironment and was associated with sensitivity to immunotherapy and chemotherapy. In in vitro experiments, silencing its expression significantly suppressed the malignant biological behavior of glioma cells, which is consistent with previous study reported.^73^ Its knockdown also notably inhibited the growth of glioma in vivo. The regulation of NF‐κB and ERK1/2 levels in the TGF‐β signaling pathway might be the molecular mechanism by which it affected glioma biology. The effects of TGF‐β on the immune and tumor microenvironment are mediated by NF‐κB and ERK 1/2, which are important regulators of immunoreaction and critical mediators in cancer initiation and progression.^74^, ^75^, ^76^ In malignancies, NF‐κB activates survival genes within cancer cells and apparently promotes the infiltration of tumor‐associated macrophages.^77^, ^78^ The ERK1/2 activation in cancer cells induces immune escape and immune resistance, driving PD‐L1 expression in malignant cells.^79^ These align with our results.

## 5 CONCLUSIONS

To summarize, we established a lncRNA signature related to TGF‐β signaling in glioma, which has significant clinical value in prognostic judgment and immunotherapy response prediction. This study broadens our comprehension of the TGF‐β signaling network in glioma and also provides a fresh outlook on the targeted regulation of TGF‐β pathways.

## AUTHOR CONTRIBUTIONS

ZX and QC conceived and designed the research. ZX, QC, WD, and LY analyzed the data. LY and JL performed animal experiments. LY performed in vitro experiments. WD, ZD, ZW, HZ, NZ, and XZ drafted the manuscript. JZ, XL, PL, ZL, HM, and CQ *contributed to* background investigation and data collection. All authors contributed to manuscript revision, read, and approved the submitted version.

## CONFLICT OF INTEREST STATEMENT

The authors report there are no competing interests to declare.

## Supporting information


Figure S1.

Figure S2.

Figure S3.

Figure S4.

Figure S5.

Figure S6.

Table S1.

Table S2.

Table S3.

Table S4.

Table S5.

## Data Availability

Data used in this work can be acquired from the TCGA and the CGGA database and so on. Furthermore, original data inquiries can be directed to the corresponding authors.
