# Supplementary figures and images for: A TGF‐β signaling‐related lncRNA signature for prediction of glioma prognosis, immune microenvironment, and immunotherapy response
Source: CNS Neurosci Ther. 2023 Oct 18;30(4):e14489. doi: 10.1111/cns.14489 (PMC11017415; doi:10.1111/cns.14489)

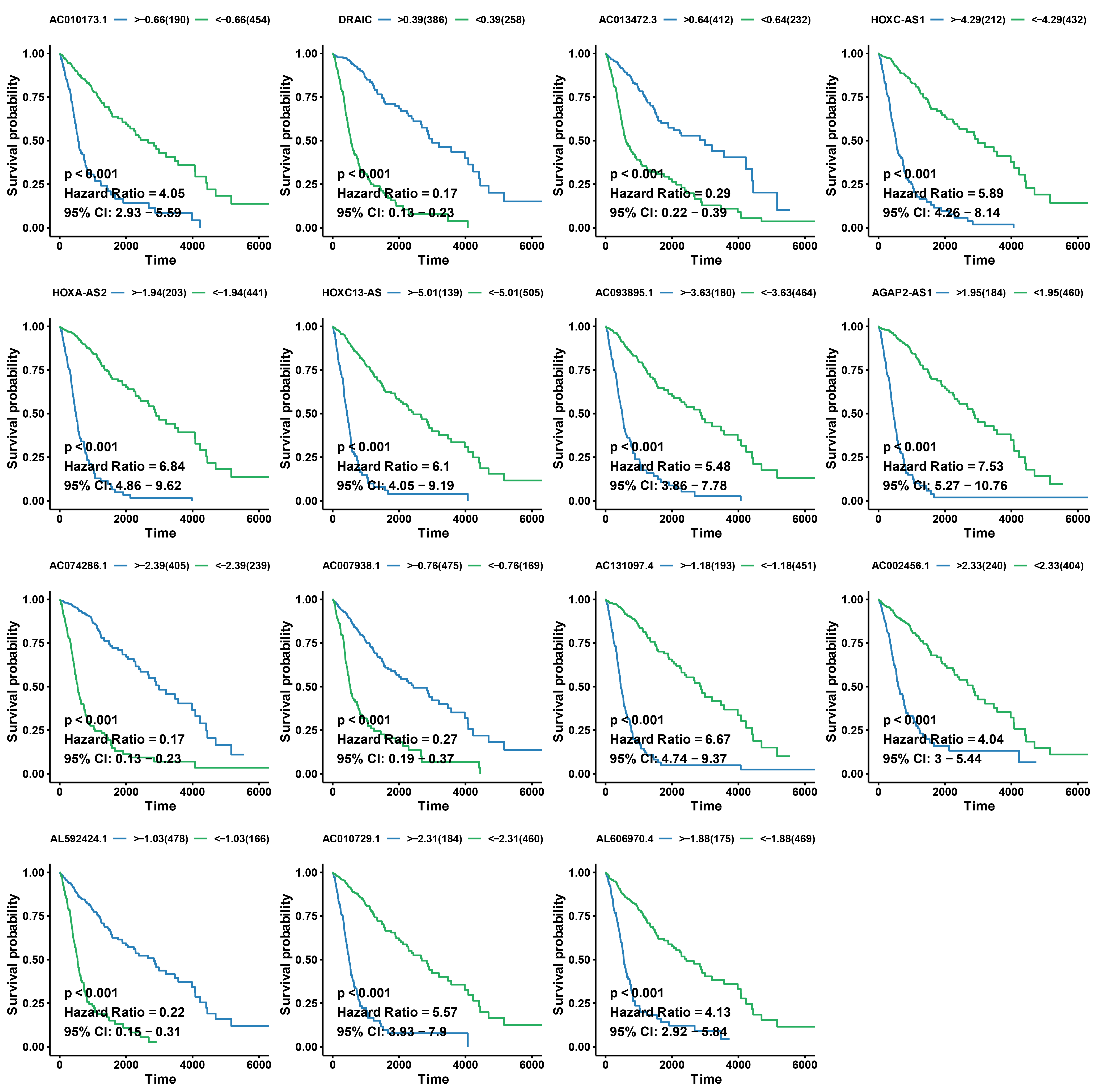

Supplement: Supplementary file 1 — Figure S1. [file CNS-30-e14489-s010.tif]

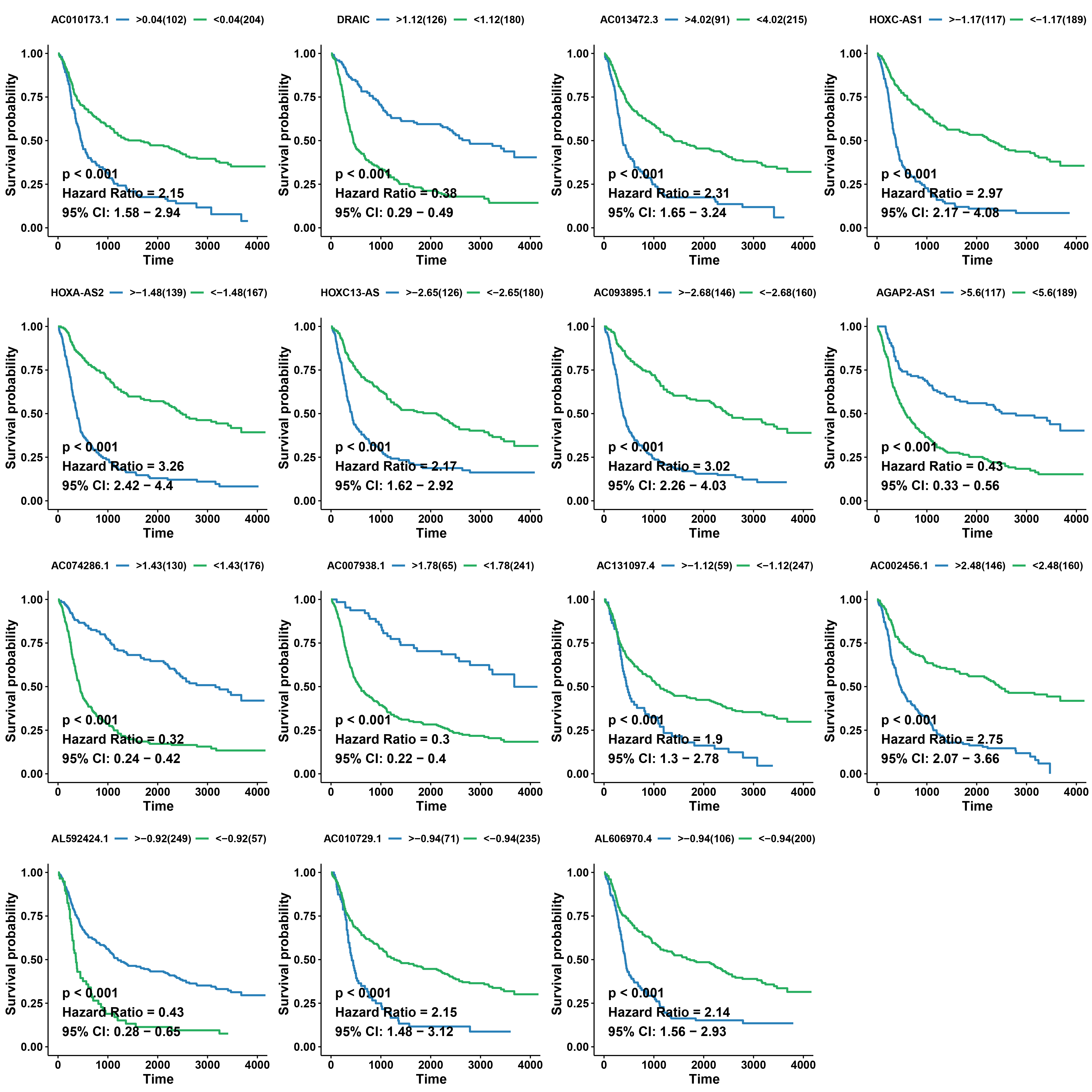

Supplement: Supplementary file 2 — Figure S2. [file CNS-30-e14489-s008.tif]

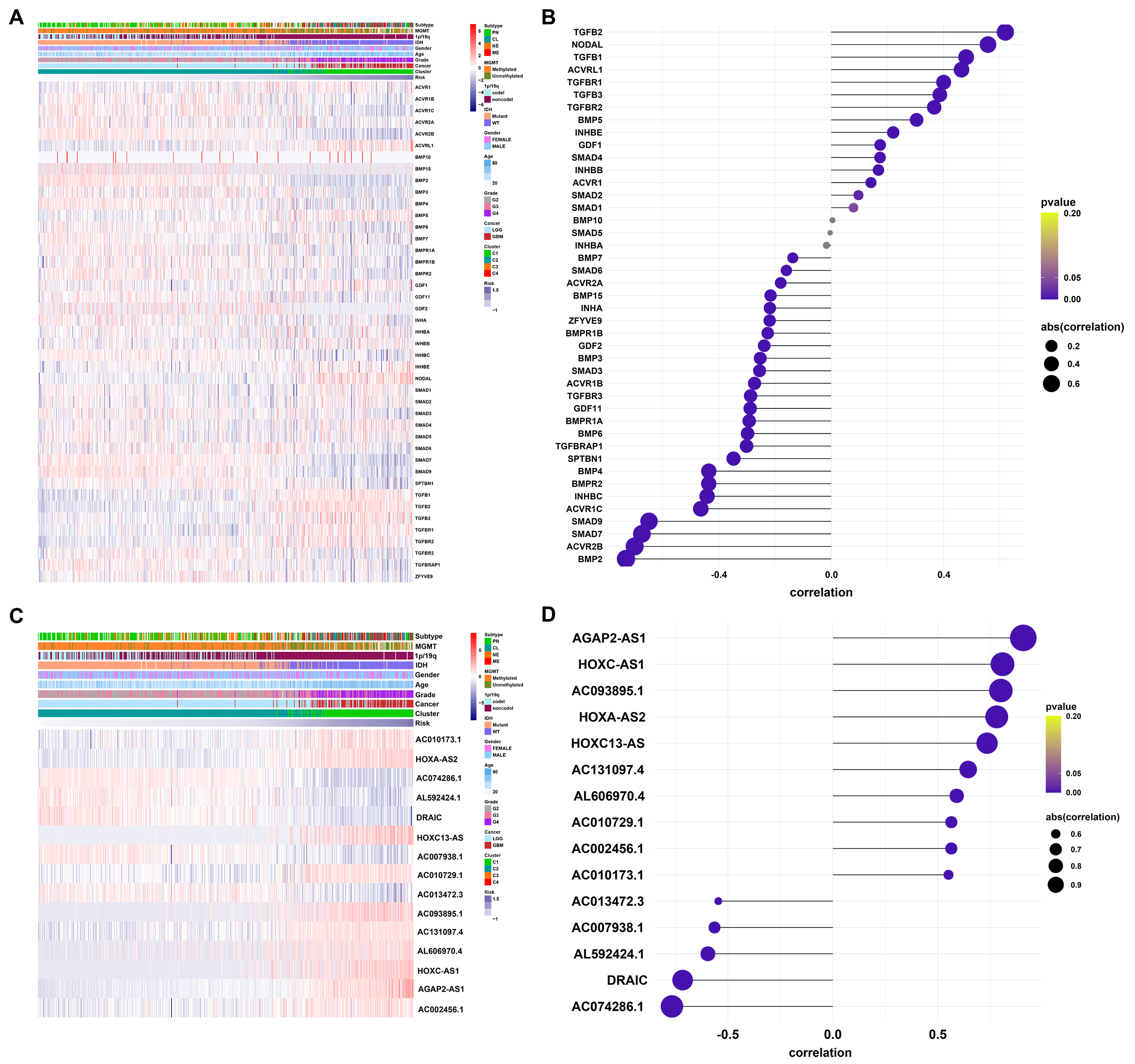

Supplement: Supplementary file 4 — Figure S4. [file CNS-30-e14489-s003.tif]

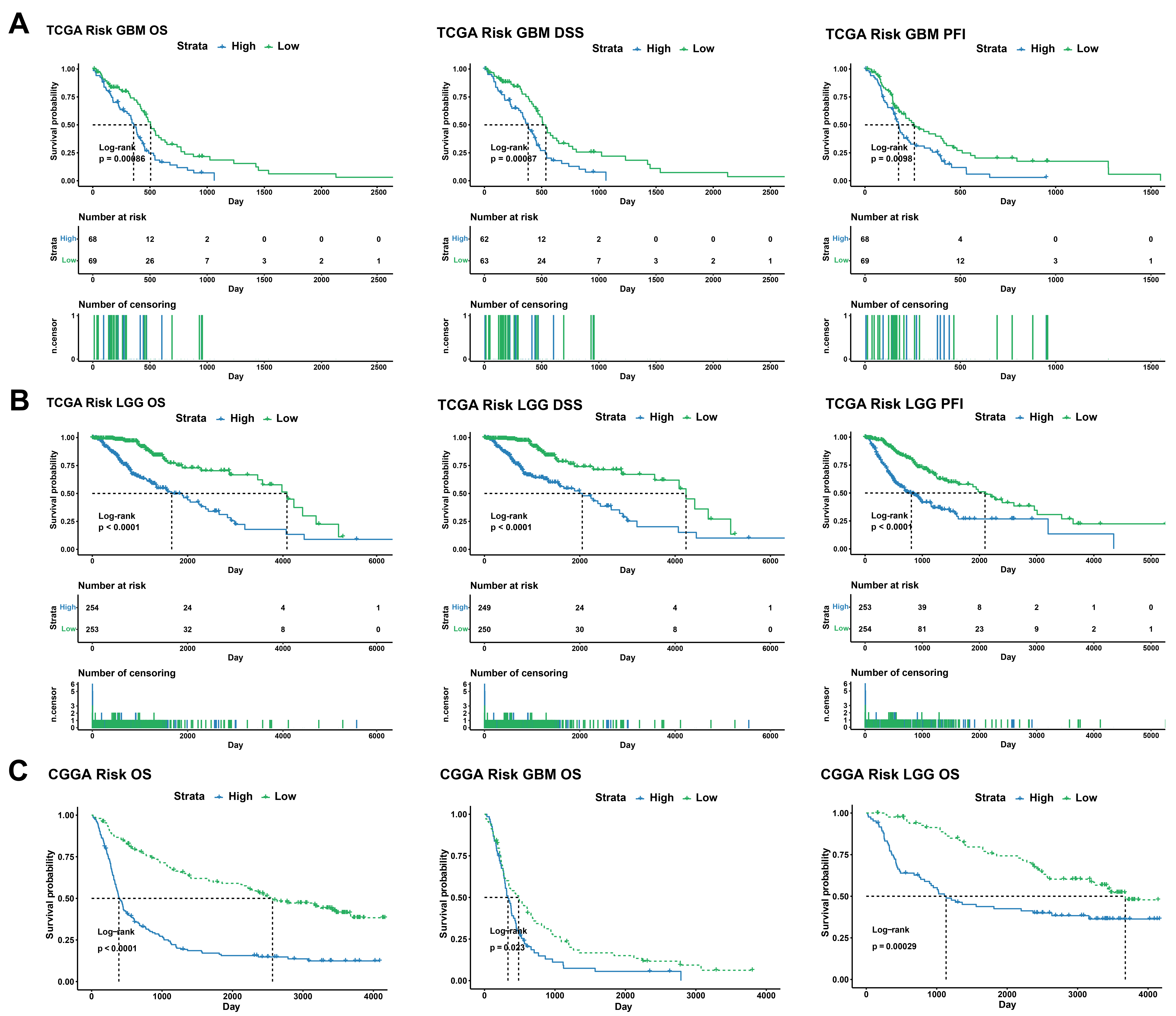

Supplement: Supplementary file 5 — Figure S5. [file CNS-30-e14489-s006.tif]

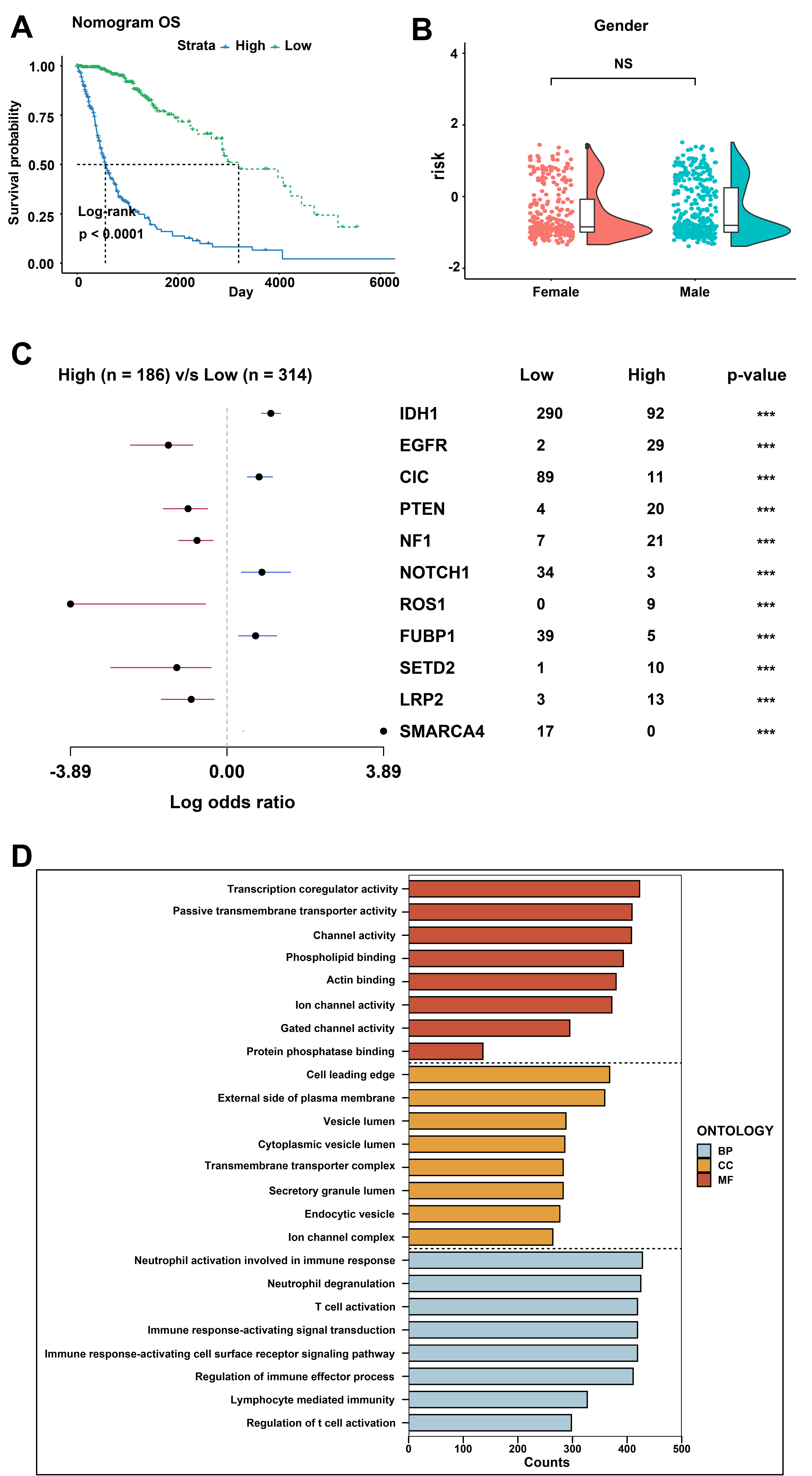

Supplement: Supplementary file 6 — Figure S6. [file CNS-30-e14489-s007.tif]
