# Supplementary material for: A TGF‐β signaling‐related lncRNA signature for prediction of glioma prognosis, immune microenvironment, and immunotherapy response
Source: CNS Neurosci Ther. 2023 Oct 18;30(4):e14489. doi: 10.1111/cns.14489 (PMC11017415; doi:10.1111/cns.14489)
Supplement: Supplementary file 7 — Table S1. [file CNS-30-e14489-s011.docx]

**List of abbreviations for 33 cancer types**

| ACC | adrenocortical carcinoma |
| --- | --- |
| BLCA | bladder urothelial carcinoma |
| BRCA | breast invasive carcinoma |
| CESC | cervical squamous cell carcinoma and endocervical adenocarcinoma |
| CHOL | cholangiocarcinoma |
| COAD | colon adenocarcinoma |
| DLBC | lymphoid neoplasm diffuse large B-cell lymphoma |
| ESCA | esophageal carcinoma |
| GBM | glioblastoma |
| HNSC | head and neck squamous cell carcinoma |
| KICH | kidney chromophobe |
| KIRC | kidney renal clear cell carcinoma |
| KIRP | kidney renal papillary cell carcinoma |
| LAML | acute myeloid leukemia |
| LGG | low grade glioma |
| LIHC | liver hepatocellular carcinoma |
| LUAD | lung adenocarcinoma |
| LUSC | lung squamous cell carcinoma |
| MESO | mesothelioma |
| OV | ovarian serous cystadenocarcinoma |
| PAAD | pancreatic adenocarcinoma |
| PCPG | pheochromocytoma and paraganglioma |
| PRAD | prostate adenocarcinoma |
| READ | rectum adenocarcinoma |
| SARC | sarcoma |
| SKCM | skin cutaneous melanoma |
| STAD | stomach adenocarcinoma |
| TGCT | testicular germ cell tumors |
| THCA | thyroid carcinoma |
| THYM | thymoma |
| UCEC | uterine corpus endometrial carcinoma |
| UCS | uterine carcinosarcoma |
| UVM | uveal melanoma |
