# Supplementary material for: A TGF‐β signaling‐related lncRNA signature for prediction of glioma prognosis, immune microenvironment, and immunotherapy response
Source: CNS Neurosci Ther. 2023 Oct 18;30(4):e14489. doi: 10.1111/cns.14489 (PMC11017415; doi:10.1111/cns.14489)
Supplement: Supplementary file 8 — Table S2. [file CNS-30-e14489-s001.docx]

**Table S2.** The sequences of siRNAs.

| siRNA-1 | CTGCCTTTCCCAAGACCGT |
| --- | --- |
| siRNA-2 | TTCTTAAACTACCAAAGGA |
| siRNA-3 | CTTGTTACCTGCTTTATAA |

Note: siRNA, small-interfering RNA.
