# Supplementary material for: A TGF‐β signaling‐related lncRNA signature for prediction of glioma prognosis, immune microenvironment, and immunotherapy response
Source: CNS Neurosci Ther. 2023 Oct 18;30(4):e14489. doi: 10.1111/cns.14489 (PMC11017415; doi:10.1111/cns.14489)
Supplement: Supplementary file 9 — Table S3. [file CNS-30-e14489-s009.docx]

**Table S3.** TGF-β signaling pathway core genes.

| ACVR1 | ACVR2B | BMP3 | BMPR1A | GDF2 | INHBE | SMAD4 | SPTBN1 | TGFBR2 |
| --- | --- | --- | --- | --- | --- | --- | --- | --- |
| ACVR1B | ACVRL1 | BMP4 | BMPR1B | INHA | NODAL | SMAD5 | TGFB1 | TGFBR3 |
| ACVR1C | BMP10 | BMP5 | BMPR2 | INHBA | SMAD1 | SMAD6 | TGFB2 | TGFBRAP1 |
| ACVR2A | BMP15 | BMP6 | GDF1 | INHBB | SMAD2 | SMAD7 | TGFB3 | ZFYVE9 |
| TGFBR1 | BMP2 | BMP7 | GDF11 | INHBC | SMAD3 | SMAD9 |  |  |
