# Supplementary material for: A TGF‐β signaling‐related lncRNA signature for prediction of glioma prognosis, immune microenvironment, and immunotherapy response
Source: CNS Neurosci Ther. 2023 Oct 18;30(4):e14489. doi: 10.1111/cns.14489 (PMC11017415; doi:10.1111/cns.14489)
Supplement: Supplementary file 10 — Table S4. [file CNS-30-e14489-s004.docx]

**Table S4.** Univariate Cox regression analysis of risk score in TCGA dataset.

|  | HR | HR.95L | HR.95H | p-value |
| --- | --- | --- | --- | --- |
| Risk (High) | 4.66 | 3.85 | 5.63 | 1.15E-56 |
| Grade (G3) | 3.58 | 2.35 | 5.47 | 3.06E-09 |
| Grade (G4) | 18.30 | 11.85 | 28.25 | 2.89E-39 |
| Age | 1.06 | 1.05 | 1.07 | 5.14E-30 |
| IDH-wildtype | 8.39 | 6.21 | 11.34 | 1.33E-43 |
| Subtype (ME) | 1.20 | 0.84 | 1.72 | 0.32 |
| Subtype (NE) | 0.20 | 0.13 | 0.32 | 6.79E-12 |
| Subtype (PN) | 0.17 | 0.12 | 0.24 | 1.34E-20 |
| 1p/19q (non-codeletion) | 4.57 | 2.91 | 7.18 | 4.51E-11 |
