# Supplementary material for: A TGF‐β signaling‐related lncRNA signature for prediction of glioma prognosis, immune microenvironment, and immunotherapy response
Source: CNS Neurosci Ther. 2023 Oct 18;30(4):e14489. doi: 10.1111/cns.14489 (PMC11017415; doi:10.1111/cns.14489)
Supplement: Supplementary file 11 — Table S5. [file CNS-30-e14489-s002.docx]

**Table S5.** Multivariate Cox regression analysis of risk score in TCGA dataset.

|  | HR | HR.95L | HR.95H | p-value |
| --- | --- | --- | --- | --- |
| Risk (High) | 2.88 | 1.88 | 4.41 | 1.07E-06 |
| Grade (G3) | 2.21 | 1.41 | 3.48 | 0.0006 |
| Grade (G4) | 2.56 | 1.43 | 4.59 | 0.002 |
| Age | 1.04 | 1.02 | 1.05 | 8.99E-08 |
| IDH-wildtype | 0.93 | 0.48 | 1.81 | 0.83 |
| 1p/19q (non-codeletion) | 1.77 | 1.03 | 3.03 | 0.04 |
| Subtype (ME) | 1.49 | 1.04 | 2.15 | 0.03 |
| Subtype (NE) | 1.73 | 0.97 | 3.09 | 0.06 |
| Subtype (PN) | 1.38 | 0.84 | 2.27 | 0.21 |
